# India's National Action Plan on Climate Change

**DOI:** 10.4103/0019-5278.50718

**Published:** 2009-04

**Authors:** Harshal T. Pandve

**Affiliations:** Department of Community Medicine, Dr. D. Y. Patil Medical College, Pimpri, Pune - 411 018, India

**Keywords:** Climate change, India, National Action Plan on Climate Change

## Abstract

Climate change is one of the most critical global challenges of our times. Recent events have emphatically demonstrated our growing vulnerability to climate change. Climate change impacts will range from affecting agriculture – further endangering food security – to sea-level rise and the accelerated erosion of coastal zones, increasing intensity of natural disasters, species extinction, and the spread of vector-borne diseases. India released its much-awaited National Action Plan on Climate Change (NAPCC) to mitigate and adapt to climate change on June 30, 2008, almost a year after it was announced. The NAPCC runs through 2017 and directs ministries to submit detailed implementation plans to the Prime Minister's Council on Climate Change by December 2008. This article briefly reviews the plan and opinion about it from different experts and organizations.

## INTRODUCTION

Climate change is one of the most critical global challenges of our times. Recent events have emphatically demonstrated our growing vulnerability to climate change. Climate change impacts will range from affecting agriculture – further endangering food security – to sea-level rise and the accelerated erosion of coastal zones, increasing intensity of natural disasters, species extinction, and the spread of vector-borne diseases.[1]

## THE PLAN IN BRIEF

The action plan outlines a number of steps to simultaneously advance India's development and climate change-related objectives. The National Action Plan on Climate Change (NAPCC) encompasses a range of measures. It focuses on eight missions, which are as follows[2]:

1. National Solar Mission: The NAPCC aims to promote the development and use of solar energy for power generation and other uses, with the ultimate objective of making solar competitive with fossil-based energy options. It also includes the establishment of a solar research center, increased international collaboration on technology development, strengthening of domestic manufacturing capacity, and increased government funding and international support.
2. National Mission for Enhanced Energy Efficiency: The NAPCC recommends mandating specific energy consumption decreases in large energy-consuming industries, with a system for companies to trade energy-saving certificates, financing for public–private partnerships to reduce energy consumption through demand-side management programs in the municipal, buildings, and agricultural sectors, and energy incentives, including reduced taxes on energy-efficient appliances.
3. National Mission on Sustainable Habitat: The NAPCC also aims at promoting energy efficiency as a core component of urban planning by extending the existing Energy Conservation Building Code, strengthening the enforcement of automotive fuel economy standards, and using pricing measures to encourage the purchase of efficient vehicles and incentives for the use of public transportation. The NAPCC also emphasizes on waste management and recycling.
4. National Water Mission: The NAPCC sets a goal of a 20% improvement in water use efficiency through pricing and other measures to deal with water scarcity as a result of climate change.
5. National Mission for Sustaining the Himalayan Ecosystem: This particular mission sets the goal to prevent melting of the Himalayan glaciers and to protect biodiversity in the Himalayan region.
6. Green India Mission: The NAPCC also aims at afforestation of 6 million hectares of degraded forest lands and expanding forest cover from 23 to 33% of India's territory.
7. National Mission for Sustainable Agriculture: The NAPCC aims to support climate adaptation in agriculture through the development of climate-resilient crops, expansion of weather insurance mechanisms, and agricultural practices.
8. National Mission on Strategic Knowledge for Climate Change: To gain a better understanding of climate science, impacts, and challenges, the plan envisions a new Climate Science Research Fund, improved climate modeling, and increased international collaboration. It also encourages private sector initiatives to develop adaptation and mitigation technologies through venture capital funds.

The NAPCC also describes other ongoing initiatives that are as follows[1]:

1. Power generation: The government is mandating the retirement of inefficient coal-fired power plants and supporting the research and development of Integrated Gasification Combined Cycle IGCC and supercritical technologies.
2. Renewable energy: Under the Electricity Act 2003 and the National Tariff Policy 2006, the central and the state electricity regulatory commissions must purchase a certain percentage of grid-based power from renewable sources.
3. Energy efficiency: Under the Energy Conservation Act 2001, large energy-consuming industries are required to undertake energy audits and an energy-labeling program for appliances has been introduced.
4. Proposals for health sector: The proposed program comprises two main components, namely provision of enhanced public health care services and assessment of increased burden of diseases due to climate change.
5. Implementation: Ministries with lead responsibility for each of the missions are directed to develop objectives, implementation strategies, timelines, and monitoring and evaluation criteria to be submitted to the Prime Minister's Council on Climate Change. The Council will also be responsible for periodically reviewing and reporting on each mission's progress. To be able to quantify progress, appropriate indicators and methodologies will be developed to assess both avoided emissions and adaptation benefits.[2]

## DIFFERENT OPINIONS ABOUT NAPCC

Mixed opinions were mentioned by the different organizations and experts over the NAPCC.

Sunita Narain, Director of Centre for Science and Environment, in an editorial in Down to Earth (a science and environment fortnightly) mentioned that the plan asserts that India can grow differently because it is in an early stage of development. In other words, it can leapfrog to a low carbon economy using high-end and emerging technologies and by being different. Also, it prioritizes national action by setting out eight missions – ranging from solar to climate research – which will be detailed and then monitored by the PM's council for climate change. But, the plan is weak on how India sees the rest of the world in this extraordinary crisis. Climate change is a global challenge. We did not create it and, till date, we contribute little to global emissions. We are, in fact, climate victims.[3]

As per Sudhirendar Sharma, a water expert and Director of the Delhi-based Ecological Foundation, the plan report is a compilation of listless ideas that lack depth, vision, and urgency. Putting economic development ahead of emission reduction targets, the report makes a case for the right of emerging economies to pursue development and growth to alleviate poverty without having to worry about the volume of atmospheric emissions they generate in the process. Consequently, the report makes no commitment to cut the country's carbon emission and thereby leaves it liable to criticism by those who hold worries about global warming close to their chests.[4]

Rahul Goswami, an independent journalist and researcher based in Goa, in his article stated that instead of having a strongly articulated, clearly thought-through vision, the NAPCC has a basket of eight “missions” and no durable plan that will include the poorest and most vulnerable A policy that deals with a new set of circumstances and factors needs necessarily to think differently. Climate change is not population control, not poverty, not rural unemployment. It needs to learn differently from the experiences of contemporary Indians.[5]

According to an article published in the Indian Express (an English daily), India has decided to stick to the safe path on dealing with climate change. One of the main reasons for taking this safe path is India's stance in multilateral negotiations. India has maintained that it believes in “common and differentiated responsibility” and hence will wait for developed countries to cap their emissions that are several times higher.[6]

Greenpeace regarded the focus on solar energy as the highlight of the plan. The solar and renewable programs showed foresight in energy planning and an intention to capitalize on the country's potential for solar energy, said the organization. But Greenpeace, in a press release, also mentioned that on the energy-efficiency front, the plan is both unambitious and vague about what the country is setting out to achieve. The Federation of Indian Chambers of Commerce and Industry (FICCI) has supported the plan, saying that eight missions will be important to leverage key initiatives that industry and others are undertaking. The market-based mechanism for energy efficiency across industry sectors will be a great driver for energy savings, according to the FICCI.[7]

WWF-India feels that the National Action Plan is fairly comprehensive in its coverage and has cross-sectoral links through the eight National Level Missions. At the centerstage of the Action Plan is India's impetus on following on a low carbon energy path without impending economic growth and quality of life of people. WWF-India feels that the Plan brings a balanced perspective on mitigation and adaptation through some new dimensions. Creation of National Mission on Strategic Knowledge for Climate Change is another good initiative as this would ensure exchange of knowledge and informed research in India.[8]

To conclude, it is now clear that initiatives to prevent climate change are started but, most importantly, these initiatives must be continuous and sustainable and every individual of every country will need to contribute to prevent climate change.[9] By releasing the NAPCC, the government has shown India's commitment to address climate change issues and also sent a positive message to the public, industries, and civil society about the government's concern to address the climate change issue through concerted action. Issues related to the awareness regarding global warming and climate change among the general population[10] and the issue related to agriculture and health hazards due to climate change must be addressed strongly and effectively.
